# Meditation in Motion: Sport Type and Meditation Level Shape Gut Microbiota Profiles in Aikido and Tai Chi Practitioners

**DOI:** 10.3390/microorganisms14020275

**Published:** 2026-01-24

**Authors:** Tehreema Ghaffar, Veronica Volpini, Francesca Ubaldi, Vincenzo Romano Spica, Federica Valeriani

**Affiliations:** Department of Movement, Human, and Health Sciences, University of Rome “Foro Italico”, 00135 Rome, Italy; t.ghaffar@studenti.uniroma4.it (T.G.); v.volpini@studenti.uniroma4.it (V.V.); f.ubaldi@studenti.uniroma4.it (F.U.); vincenzo.romanospica@uniroma4.it (V.R.S.)

**Keywords:** meditation, sport, gut microbiota, aikido, taichi, gut–brain

## Abstract

Mind–body practices integrating movement and meditation, such as Tai Chi and Aikido, have been proposed to influence the gut–brain axis through combined physiological and psychological pathways. However, evidence regarding their association with gut microbiota composition remains limited. This study explored gut microbiota diversity and taxonomic profiles in regular practitioners of Tai Chi and Aikido across different levels of meditation depth. Forty-two adults practicing Tai Chi or Aikido provided fecal samples for 16S rRNA sequencing, and meditation depth was assessed using the Meditation Depth Questionnaire (MEDEQ). Alpha diversity did not differ significantly between groups, although a descriptive trend toward higher diversity with increasing meditation depth was observed. Beta-diversity analyses suggested compositional differences associated with meditation level (ANOSIM R = 0.191, *p* = 0.035), along with an exploratory interaction signal between practice type and meditation depth (ANOSIM R = 0.296, *p* = 0.001). Taxonomic profiling highlighted distinct microbial patterns associated with both practice type and meditation depth. Short-chain fatty acid-associated genera, including *Faecalibacterium* and *Roseburia*, were relatively more abundant in Aikido practitioners with higher meditation scores, whereas Tai Chi practitioners showed higher relative abundances of *Prevotella* and *Collinsella*. Overall, these findings indicate that meditative movement practices are associated with distinct gut microbiota compositional patterns within this cohort. Given the exploratory and cross-sectional design, these results should be interpreted as hypothesis-generating. Future longitudinal studies incorporating functional and clinical outcomes are needed to clarify underlying mechanisms.

## 1. Introduction

In an era dominated by digital engagement and increasingly sedentary habits, depression has emerged as one of the most widespread mental health conditions of the 21st century, affecting over 280 million individuals worldwide [1]. Contemporary lifestyles are marked not only by insufficient physical activity but, in specific populations, also by excessive or performance-oriented training [2,3,4]. Both physical inactivity and excessive exercise have been linked to physiological stress and dysregulation of metabolic, immune, and neuroendocrine pathways, underscoring the importance of balanced, integrative movement-based lifestyle approaches [2,3,4,5].

Insufficient physical activity is consistently linked to increased risk of chronic dis-ease, low-grade inflammation, and depressive symptoms [6,7,8,9]. Conversely, evidence de-rived largely from competitive athletic populations indicates that prolonged high-intensity training may induce physiological stress, impair immune function, and disrupt gut barrier integrity [10]. Although physical activity per se modulates gut microbiota composition in-dependently of diet across diverse populations [11], more pronounced gastrointestinal disturbances and stress-related microbial alterations are predominantly observed in endurance and performance-oriented athletes exposed to high training loads [12]. These findings highlight that adverse physiological adaptations may arise at both ends of the activity continuum. Despite the well-established benefits of physical activity across the life course, global levels of inactivity remain high, with nearly one-third of adults failing to meet recommended activity levels [1,13,14,15]. This persistent gap between public health recommendations and real-world behaviors has reinforced interest in accessible, sustainable, and holistic forms of movement that integrate physical and mental dimensions [1,16].

Within this context, the gut microbiota has emerged as a critical mediator connecting lifestyle behaviors with physical and mental well-being [17]. This diverse microbial ecosystem interacts bidirectionally with physical activity, immune function, and metabolic pathways [18]. Active individuals typically exhibit higher microbial richness and more favorable microbial profiles than sedentary counterparts [19,20], contributing to improved gastrointestinal function, reduced systemic inflammation, and enhanced metabolic resilience. However, not all forms of physical activity exert the same influence on the microbiome. Practices integrating intentional movement, breath control, and sustained mental focus—often referred to as meditative movement—may modulate physiological and psychological systems simultaneously, influencing the gut–brain–microbiota axis through distinct mechanisms [21]. Physical activity also impacts the autonomic nervous system, including vagal nerve function, a key component of the gut–brain axis whose dysregulation is closely linked to depression [22,23].

Meditation alone is widely recognized for its benefits in reducing stress, improving emotional regulation, decreasing inflammation, and modulating neuroendocrine pathways [24]. Mindfulness-based practices lower cortisol, support cardiovascular regulation, and attenuate systemic inflammatory markers [25,26]. Stress is a primary mediator of gut microbial composition and intestinal permeability [27]; chronic psychological stress disrupts microbial diversity and promotes dysbiosis, contributing to gastrointestinal and mood disorders [22]. Conversely, meditation and mindfulness may contribute to a more stable gut environment, potentially through reduced inflammatory cytokine signaling and modulation of vagal tone [28,29]. These findings support the hypothesis that practices combining physical movement with meditative engagement may exert synergistic effects on both mental and gut health [28].

Martial arts represent a unique category of mind–body disciplines that integrate physical exercise, breath regulation, focus, and philosophical principles [30]. Tai Chi and Aikido, both traditionally rooted in Eastern philosophies, are soft martial arts characterized by coordinated movement, balance training, controlled breathing, and heightened body awareness [31]. These practices emphasize harmony, proprioception, and mental clarity, positioning them as paradigmatic forms of meditative movement rather than performance-driven exercise [30,31,32]. Evidence indicates that martial arts practice is associated with improvements in physical fitness, emotional regulation, psychological well-being, and social connectedness, with reductions in anxiety and depressive symptoms reported particularly in non-clinical, community-based populations [31,32,33].

Tai Chi is a low-impact internal martial art characterized by slow, flowing movements and sustained meditative awareness, with evidence suggesting benefits for mental health, balance, cardiovascular function, and immune–inflammatory regulation [34,35,36,37,38]. The available literature further indicates that Tai Chi and other martial arts may reduce depressive symptoms, particularly in the short term, with mental health benefits partly mediated by social connectedness arising from group-based practice settings, as shown by mediation analyses in intervention studies [36,37,38,39,40].

Within the spectrum of meditative martial arts, Aikido represents a distinct Japanese discipline emphasizing harmony, circular and fluid movements, breath regulation, and embodied awareness [41]. Often described as “meditation in action,” Aikido cultivates heightened attention, centeredness, and psychological calm during practice [42]. Although less extensively studied than Tai Chi, Aikido shares core elements of mind–body integration and may exert comparable influences on mental and physiological pathways, including stress-responsive and immune-related mechanisms [43,44,45].

The metabolic intensity of martial arts varies considerably: Tai Chi ranges from 1.5 to 3.0 METs (Metabolic Equivalent of Task) (light-to-moderate intensity), slower-paced Aikido averages around 5.3 METs (moderate intensity), whereas combat sports such as judo, karate, or kickboxing reach 10.3 METs (vigorous intensity) [46]. Unlike high-intensity competitive sports, meditative martial arts integrate moderate physical activity with mindfulness and self-regulation, potentially offering unique benefits for mental health [32,38,40,47,48,49]. By operating within light-to-moderate intensity ranges, they are consistent with international physical activity guidelines and may represent sustainable, accessible forms of movement across the life course [14,15,32].

Nevertheless, empirical evidence on gut microbial profiles in individuals practicing meditative martial arts remains limited [50]. While extensive research has examined the gut microbiota of competitive athletes—demonstrating sport-specific microbial adaptations driven by training intensity and physiological load [5,51,52,53,54,55]—non-competitive, meditative martial arts remain underexplored. Studies demonstrate that the type of sport, intensity of training, and physiological load differentially shape gut microbial profiles, with endurance, strength, and combat sports each presenting distinct signatures [5,54]. These findings highlight the adaptive capacity of the microbiota to the metabolic, neuroendocrine, and inflammatory demands of training [53,54,55]. However, most existing research has focused on competitive athletes engaged in high-intensity or performance-driven activities, leaving an important gap regarding non-competitive, meditative martial arts such as Tai Chi and Aikido [56,57]. Their unique combination of moderate physical activity, mindfulness, and autonomic regulation may influence the gut microbiota through mechanisms distinct from those observed in performance-oriented sports [54,55,56,57].

The present study, therefore, investigates gut microbiota composition in individuals practicing Tai Chi and Aikido, to explore whether these meditative movement practices are associated with distinct microbial profiles in relation to both sport type and depth of meditative engagement. By focusing on a population that integrates movement and meditation simultaneously, this exploratory study seeks to address an important gap in current microbiome research and to generate hypotheses for future mechanistic and longitudinal investigations.

## 2. Materials and Methods

### 2.1. Study Design and Ethical Approval

The research protocol received approval from the Institutional Review Board at the University of Rome “Foro Italico” (approval number CAR 204/2024). The study was conducted in accordance with the principles of the Declaration of Helsinki. During the recruitment process, investigators explained the aims of the study, its procedures, and the ethical safeguards in place, including data anonymity and confidentiality. Before participation, all subjects provided written informed consent.

### 2.2. Participants and Recruitment

**Figure 1:**
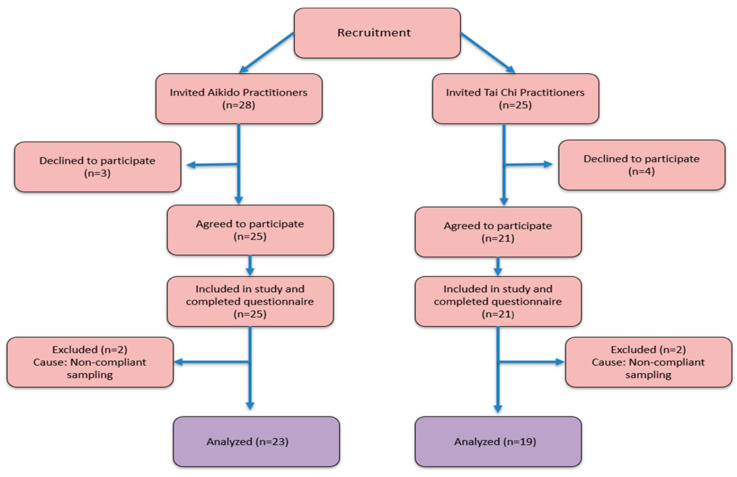
Study flowchart of participant enrollment and analysis. Diagram showing recruitment of 53 invited participants, exclusions for non-compliant sampling, and final inclusion of 42 Tai Chi and Aikido practitioners with complete questionnaire and fecal microbiota data.

A total of 53 adult practitioners of Tai Chi and Aikido were invited to participate after their regular training sessions. Of these, 46 agreed to take part and completed all study procedures, including informed consent, questionnaire completion, and biological sample collection. Four samples were excluded from the final analysis due to protocol non-compliance (e.g., improper labeling or delayed sample submission). Consequently, the final analytical sample consisted of 42 participants, as shown in Figure 1. Inclusion criteria included regular practice of Tai Chi (Tai Chi group) or Aikido (Aikido group), willingness to provide biological samples and complete questionnaires, and absence of antibiotic or probiotic use or gastrointestinal procedures within the previous three months. All participant data were anonymized using a non-identifiable alphanumeric code, consistently applied to biological samples and questionnaires. Participants were adults within an age range of 18 years and older (including individuals > 65 years), and sex distribution is summarized in Table 1. All inclusion and exclusion criteria, including antibiotic or probiotic use, recent gastrointestinal procedures, chronic diseases, pregnancy, and other medical conditions potentially affecting gut microbiota composition, were applied during the recruitment phase, prior to participant enrollment and biological sample collection.

### 2.3. Biological Sample Collection and Handling

Each participant received a fecal swab (Copan Italia S.p.A., Brescia, Italy) along with standardized instructions for fecal sample collection. A single fecal sample was collected from each participant as part of this cross-sectional study design. Participants were asked to collect a single fecal sample on a designated day and to submit the swab within two hours of collection. While a strict circadian collection window was not imposed, participants were instructed to collect samples under consistent daily conditions and to avoid collection immediately following intense physical activity. Samples were stored at 4–8 °C in a refrigerated container and transported to the laboratory at the University of Rome “Foro Italico” within 24 h, where they were processed according to a previously validated protocol for DNA extraction from fecal material.

### 2.4. Questionnaire

Participants were asked to fill out a questionnaire on the EU survey platform, which collected details about their age, gender, weight, height, and any specific dietary practices they might follow (such as vegetarian or vegan diets). It was emphasized that providing this information would not result in the exclusion of any participants. Furthermore, the presence of ongoing chronic illnesses, pregnancy, food sensitivities, concurrent infections (along with the use of antibiotics and probiotics), or gastrointestinal surgical procedures within the last three months were considered as exclusion criteria. The 9-item questionnaire created by Martínez-González et al. and the abbreviated version of the International Physical Activity Questionnaire (IPAQ) were utilized to evaluate participants’ eating habits and typical physical activity levels [58,59]. In addition, a 35-item evaluation tool known as the Meditation Depth Questionnaire (MEDEQ) was implemented to assess the intensity of meditation engaged in by each participant [60]. The opening questions of this questionnaire focus on the techniques and duration of meditation, covering both the number of years practiced and the minutes spent in each session. The latter section consists of 30 items aimed at gathering insights about the physical and mental experiences encountered by subjects during meditation. This information is then leveraged to calculate the MEDI, a quantitative metric for meditation depth. The MEDI and MEDEQ tools were both devised by Piron et al. [60]. For every question, participants were asked to indicate their degree of agreement on a scale from 0 (indicating no agreement) to 4 (indicating strong agreement). The overall score was derived by adding up the factors identified as supportive of meditation specifically factors 3, 4, 6, 7, 8, 9, 11, 12, 15, 16. The final score is reached by summing up the scores for factors 16, 17, 19–30, and items 1, 4, 9, 12, 13, and 18, which are inversely linked to meditation. It is important to highlight that the total score does not pertain to the time element, but rather to the physical and mental sensations experienced during meditation. Although the Meditation Depth Questionnaire (MEDEQ) and the International Physical Activity Questionnaire–Short Form (IPAQ-SF) have been validated in their respective original populations, they have not been specifically validated in practitioners of meditative martial arts such as Tai Chi or Aikido. In the present study, these instruments were therefore used as exploratory tools to characterize relative differences in meditation depth and general physical activity levels within the study cohort, rather than for diagnostic or comparative purposes across populations.

### 2.5. Data Collection

Given the exploratory and cross-sectional nature of the study, a formal a priori sample size calculation was not performed to power specific hypothesis-testing comparisons. The final sample size was determined by feasibility and participant availability during the recruitment period. Any previously reported power considerations based on paired designs have been removed, as they were not appropriate for the unpaired study design. Participants were recruited after an invitation presented at the end of class. Those who agreed to participate received the study materials on pre-scheduled days. The materials included an information letter, an informed consent form, written instructions, test tubes for fecal sample collection, corresponding labels, and a link to access the questionnaire. The fecal samples were collected separately, adhering to detailed guidelines, and were kept refrigerated until they were delivered on designated days and times. To maintain anonymity, each participant was assigned a code made up of the first three letters of the mother’s name and the father’s birth year (e.g., FRA1967), which remained unknown to the researchers. The identification code was affixed to each tube using the supplied labels and included in the questionnaire, allowing for cross-analysis of the data. The questionnaire, which contained both open-ended and closed-ended questions, was distributed through a link on the EU Survey platform, the official European Commission tool for conducting online surveys.

### 2.6. DNA Extraction from 16S rDNA Amplicon

A fecal swab (Copan Italia S.P.A., Brescia, Italy) was given to each participant, along with detailed instructions for stool sample collection. Participants were told to submit their samples on a specified date, within two hours of collection. The samples were kept at 4–8 °C in a refrigerated container and transported to the laboratory at the University of Rome “Foro Italico” within 24 h. Upon receipt, fecal samples were weighed, and DNA was extracted following a validated protocol for isolating microbial DNA from fecal material. The DNA was then purified and normalized before sequencing. Amplicon libraries were created targeting the V1–V3 region of the 16S rRNA gene, adhering to the 16S Metagenomic Sequencing Library Preparation Guide (part# 15044223 Rev. A; Illumina, San Diego, CA, USA) [61,62]. Tagged polymerase chain reaction (PCR) products were generated through a two-step PCR process using unique barcoded primer pairs. In this approach, the target primers containing adapters were employed in the first PCR to amplify the target gene (16S rDNA), and the resulting product was used in the second PCR with primers that included barcodes for individual sample identification. Each amplification reaction comprised a total volume of 25 μL, including 12.5 μL of KAPA HiFi Hot Start Ready Mix (Roche, Pleasanton, CA, USA), 5 μL of each primer (1 μM), and 2 μL of template deoxyribonucleic acid (DNA). Reactions were conducted on a Techne^®^ TC-PLUS thermal cycler (VWR International, LLC, Radnor, PA, USA).

### 2.7. Illumina Sequencing and Bioinformatics Analysis

The amplification is performed and 5 μL of PCR product from every reaction was analyzed through agarose gel electrophoresis (1%) to verify amplification. The final concentration of the purified DNA amplicon was measured using the Qubit PicoGreen dsDNA BR Assay Kit (Invitrogen, Grand Island, NY, USA). Libraries were prepared following the MiSeq Reagent Kit Preparation Guide (Illumina, San Diego, CA, USA) and utilized the iSeq100 sequencing platform (Illumina, San Diego, CA, USA). Raw sequencing data were processed with a customized bioinformatics pipeline based on the Galaxy platform, incorporating quality control and taxonomic assignment tools. Initial quality evaluation of FASTQ files was carried out using FASTA/Q Information Tools and Mothur. Reads were filtered to remove low-quality sequences (length < 200 nt), ambiguous base calls, and chimeric sequences. High-quality sequences were grouped into operational taxonomic units (OTUs) at 97% sequence identity, employing a de novo clustering method. Representative sequences from each OTU were classified taxonomically using the Ribosomal Database Project (RDP) Classifier (version 2.5), which assigns taxonomic ranks through a naïve Bayesian algorithm. OTUs identified at 97% similarity were regarded as species-level units, while 95% similarity was applied for genus-level classifications. Furthermore, sequencing reads were concurrently examined using the Illumina BaseSpace 16S Metagenomics App (version 1.0.1), which employs an alternative taxonomic classification pipeline based on the curated May 2013 release of the Greengenes reference database. Only taxes with a relative abundance exceeding 0.1% were included in subsequent statistical analyses.

### 2.8. Statistical Analysis

Meditation depth was assessed using the MEDI score and, for descriptive purposes, participants were categorized into three levels (low, medium, high). The MEDI reflects subjective physical and mental experiences during practice rather than meditation duration. Given the limited size and imbalance of some meditation-level strata, analyses involving meditation categories were interpreted exclusively as exploratory. Gut microbiota relative abundances were calculated at the phylum and genus levels using an OTU-based approach at 97% sequence similarity. Taxonomic assignment was performed using the RDP classifier and the Greengenes reference database. Very low-abundance phyla (<0.1% across all samples) were excluded to reduce data sparsity. Alpha diversity (Shannon index and evenness) and beta diversity were computed using PRIMER7. Normality of alpha-diversity distributions was assessed using the Shapiro–Wilk test. Group comparisons were conducted using parametric or non-parametric tests as appropriate (independent samples t-test, Mann–Whitney U test, or Kruskal–Wallis test). Genus-level richness was estimated using a Chao1-like index as an exploratory complement [63]. Beta-diversity patterns were explored using principal coordinate analysis (PCoA), and differences in community composition were assessed using analysis of similarity (ANOSIM). Given the exploratory design and baseline imbalances in sex distribution and MEDI score, these variables were considered during interpretation rather than used for confirmatory adjustment. All statistical analyses were performed using PRIMER-e Ltd. (version 7.), Python (version 3.10) with the SciPy library, and IBM SPSS Statistics version 25. Statistical significance was set at *p* < 0.05. For analytical purposes, participants were categorized according to practice type (Tai Chi vs. Aikido) and sex (male vs. female). Sex-stratified analyses were conducted within each practice group for descriptive comparison only.

## 3. Results

### 3.1. Participant Information and Study Design (General Characteristics of the Subjects)

We collected a total of 42 fecal samples from adult practitioners of two meditative martial arts, Tai Chi and Aikido (Table 1). Both practices integrate structured physical movement with meditative components. Descriptive analyses were performed for anthropometric, sociodemographic, and lifestyle variables. Body mass index (BMI) was calculated as body weight (kg) divided by height squared (m^2^). For analytical purposes, participants were categorized according to two independent factors: (i) type of meditative movement practice (Tai Chi vs. Aikido) and (ii) sex (male vs. female). Analyses examined the main effects of practice type and meditation depth, with additional sex-stratified comparisons performed within each practice group. The term “exercise + gender” refers exclusively to these sex-stratified analyses and does not represent a separate or combined experimental group. Data were collected on age, sex, BMI, educational level, smoking and alcohol habits, dietary patterns, physical activity level, meditation score, and practice level. Overall, the two groups were largely comparable across most variables; however, significant differences were observed in sex distribution and MEDI score (Table 1). Physical activity was quantified in metabolic equivalent of task (MET) units. Data are reported as mean ± standard deviation (SD), and no significant differences were detected for the remaining variables based on t-test comparisons.

### 3.2. Alpha Diversity Indices

**Figure 2:**
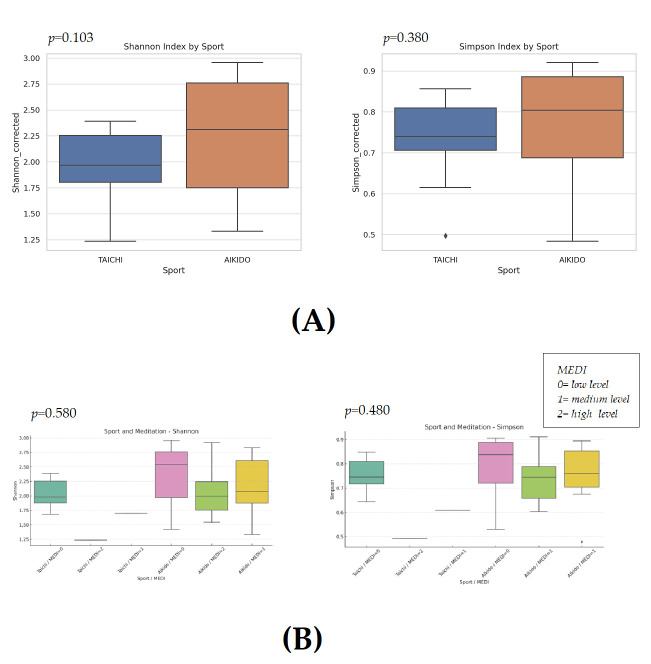

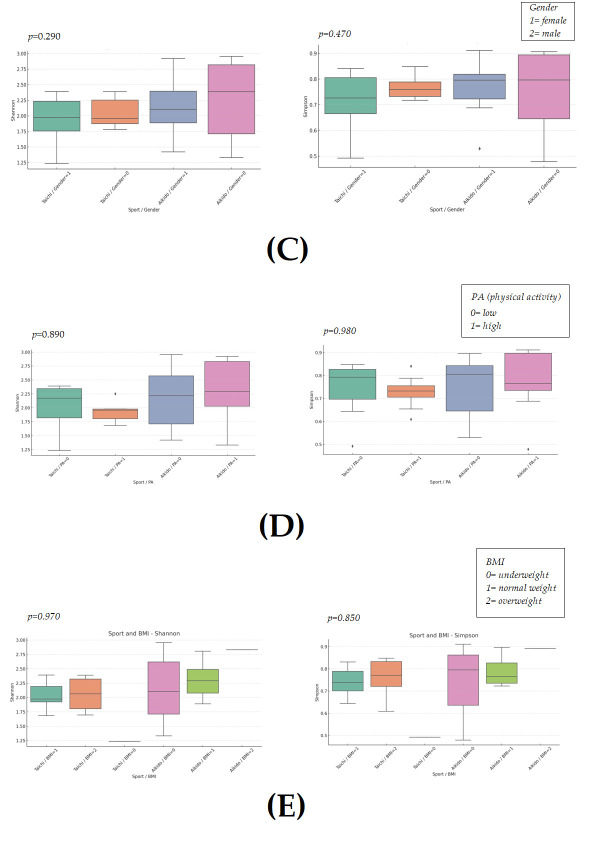
(**A**). Alpha diversity by sport type. Boxplots of Shannon and Simpson indices comparing Tai Chi and Aikido practitioners. Aikido tended toward higher diversity, although differences were not statistically significant. (**B**). Alpha diversity by meditation level. Boxplots of Shannon and Simpson indices across low, medium, and high meditation groups. No significant differences were observed, but higher meditation levels suggested a trend toward increased diversity. Meditation-level subgroups include highly imbalanced sample sizes (including *n* = 1); results are shown for descriptive purposes only. (**C**). Alpha diversity by gender. Boxplots of Shannon and Simpson indices separated by male and female participants. Diversity measures were comparable across genders. (**D**). Alpha diversity by sport level. Boxplots of Shannon and Simpson indices stratified by reported intensity or level of practice. No significant differences were detected. (**E**). Alpha diversity by BMI. Boxplots of Shannon and Simpson indices for normal weight, overweight, and underweight categories. Indices were consistent across BMI groups.

Alpha diversity analysis (Shannon and Simpson indices) was evaluated across the categories of Sport, Meditation levels, Physical activity levels, Gender and BMI using *t*-test, Mann–Whitney U test and Kruskal–Wallis test. No statistically significant differences were found in the Shannon or Simpson indices when comparing the groups (*p* > 0.05 for all comparisons). A marginally significant trend was noted for the Shannon index when examining different sports, implying a potential variation between Taichi and Aikido, although this did not reach the level of statistical significance. No significant differences in alpha diversity were observed between Aikido and Tai Chi groups (Shannon *p* = 0.103; Simpson *p* = 0.38), although the Aikido group tended to exhibit higher diversity (Figure 2A). Boxplots depicting the diversity indices highlighted these trends, with the Shannon index spanning from approximately 1.8 to 2.25 among the groups. The Simpson index showed relative stability, ranging from about 0.71 to 0.81. When considering additional factors, stratified by Meditation level, no significant trends were observed in alpha diversity (Kruskal–Wallis test; Shannon: *p* = 0.58; Simpson: *p* = 0.48). Diversity indices remained comparable across low, medium, and high meditation groups. For Taichi + Meditation levels 1 and 2, only one sample was available for each (*n* = 1), which limited the statistical power in these specific subgroups (Figure 2B). Moreover, no significant differences were found, the visual patterns were visually observed that the type of sport might affect diversity indices. No significant differences were detected between male and female participants (Shannon: *p* = 0.29; Simpson: *p* = 0.47), indicating that gender did not influence gut microbial diversity in this cohort (Figure 2C). Analysis of diversity across Physical Activity levels yielded no significant differences (Shannon: *p* = 0.89; Simpson: *p* = 0.98); both lower and higher PA groups displayed overlapping ranges of diversity indices (Figure 2D). Similarly, BMI categories like underweight, normal weight, overweight (Kruskal–Wallis test; Shannon: *p* = 0.97; Simpson: *p* = 0.85) did not show significant effects on alpha diversity (Figure 2E).

Overall alpha diversity (Shannon and Simpson) was not significantly different between Aikido and Tai Chi participants, although a tendency toward higher diversity was observed in the Aikido group (Mann–Whitney U test: Shannon *p* = 0.103; Simpson *p* = 0.38). Among other variables, higher alpha diversity values were observed across increasing meditation levels. Given the very small size of some meditation-level subgroups, these observations are presented for descriptive purposes only and should be interpreted with caution. As an exploratory complement to Shannon and Simpson indices, genus-level richness was estimated using a Chao1-like index (mean ± SD: 189.7 ± 50.3; range: 103.6–287.5). Chao1 estimates were consistent with patterns observed for the other alpha-diversity indices and did not materially affect the interpretation of the results.

### 3.3. Beta Diversity and Compositional Differences (PCoA)

**Figure 3:**
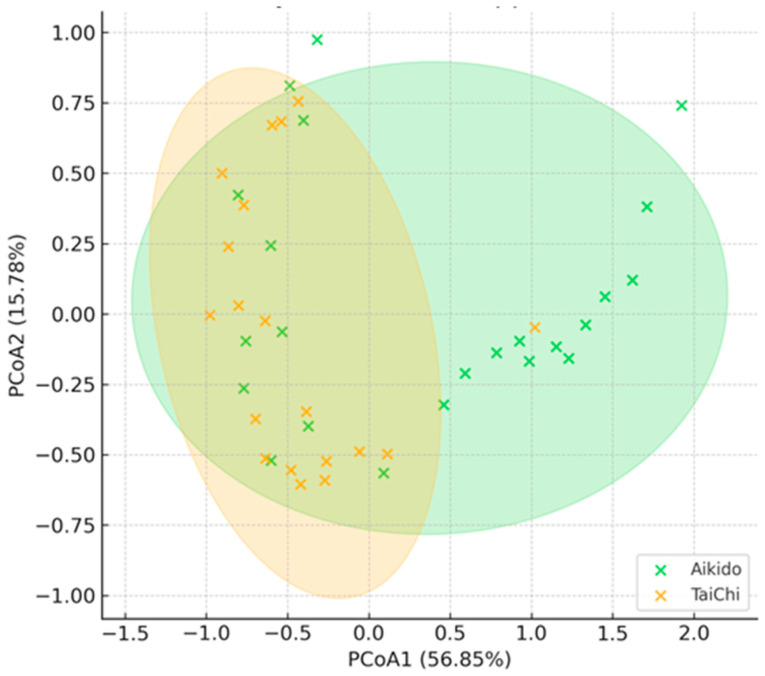
Beta diversity by sport type. Principal Coordinates Analysis (PCoA) of Bray–Curtis distances showing partial separation of Tai Chi and Aikido microbiota compositions with 95% confidence ellipses.

Beta diversity was assessed using Bray–Curtis dissimilarity and represented through Principal Coordinates Analysis (PCoA). The initial two PCoA axes accounted for 56.85% and 15.78% of the overall variation, respectively. Clear separation patterns were noted between the Aikido and Tai Chi groups, indicating differences in their microbiota composition (Figure 3). Confidence ellipses (95%) were incorporated to illustrate the dispersion at the group level. In this group of Aikido and Taichi practitioners, the level of Meditation was found to be the most significant factor impacting the structure of the gut microbial community. Notable differences in beta diversity were detected across varying Meditation levels (R = 0.191–0.240, *p* < 0.05), with the strongest exploratory signal observed for the Sport × Meditation interaction (R = 0.296, *p* = 0.001), indicating that the microbiota response to meditation is influenced by the type of sport practiced. However, this finding should be interpreted with caution due to the very small size of some meditation-level strata and is intended as hypothesis-generating. A slight interaction between Sport and Gender was also identified (R = 0.091, *p* = 0.049). On the other hand, Sport type alone, BMI, Physical Activity level, and Gender did not have a significant impact on beta diversity. These results underscore the potential influence of meditative practices on gut microbiota within this active cohort.

**Figure 4:**
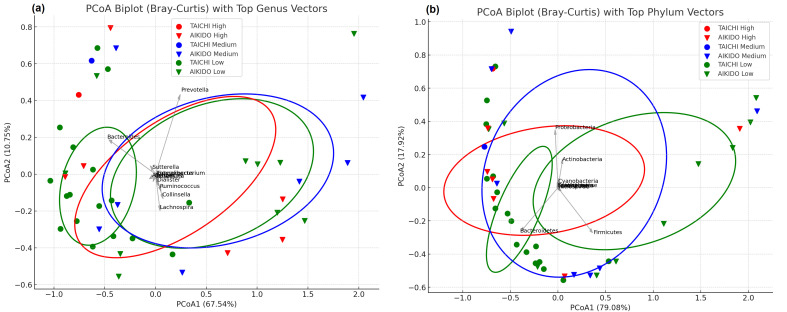
Beta diversity at genus and phylum levels. (**a**). PCoA plots based on Bray–Curtis distances at the genus level, highlighting differences in microbial composition between Tai Chi and Aikido participants. (**b**). PCoA based on Bray–Curtis distances at the phylum level, showing the contribution of dominant phyla (e.g., *Firmicutes* and *Bacteroidetes*) to sample separation.

At the level of genus (Figure 4a), PCoA1 and PCoA2 accounted for 67.54% and 10.75% of the overall variance, respectively. Samples from Aikido and Tai Chi showed some degree of separation, although there was also considerable overlap. The primary genera influencing this distinction included: *Prevotella* (more abundant in Tai Chi); *Bacteroides* (more abundant in Aikido); along with *Sutterella*, *Ruminococcus*, *Collinsella*, *Dialister*, and *Lachnospira*. This indicates that the community structure at the genus level varies moderately between the two groups, with certain taxa responsible for driving these variations. At the phylum level (Figure 4b), PCoA1 and PCoA2 represented 79.08% and 17.92% of the variance. A more distinct separation between Aikido and Tai Chi samples was noted. The key contributors to this separation included *Firmicutes* (more prevalent in Taichi) and *Bacteroidetes* (more prevalent in Aikido), along with additional contributions from *Proteobacteria*, *Actinobacteria*, and *Cyanobacteria*. These results highlight that the composition of gut microbiota at the phylum level more effectively differentiates between practitioners of Aikido and Tai Chi than at the genus level. Collectively, these analyses reveal that both genus and phylum level compositions of microbiota differ between Aikido and Tai Chi groups, with the most significant variations linked to changes in the relative abundance of *Firmicutes* and *Bacteroidetes*.

The Analysis of Similarities (ANOSIM) indicated notable differences in the composition of gut microbiota across varying levels of meditation and between groups defined by sport type and meditation level (Table S1). The most pronounced separation was found in the combined TYPE + LEVEL MEDI comparison (R = 0.296, *p* = 0.001), suggesting that the type of sport and the level of meditation together influence distinct microbial profiles. Additionally, significant differences were noted in both gender-specific comparisons and overall meditation level assessments.

### 3.4. Gut Microbiota Composition Differences Based on Meditation Levels & Gender

**Figure 5:**
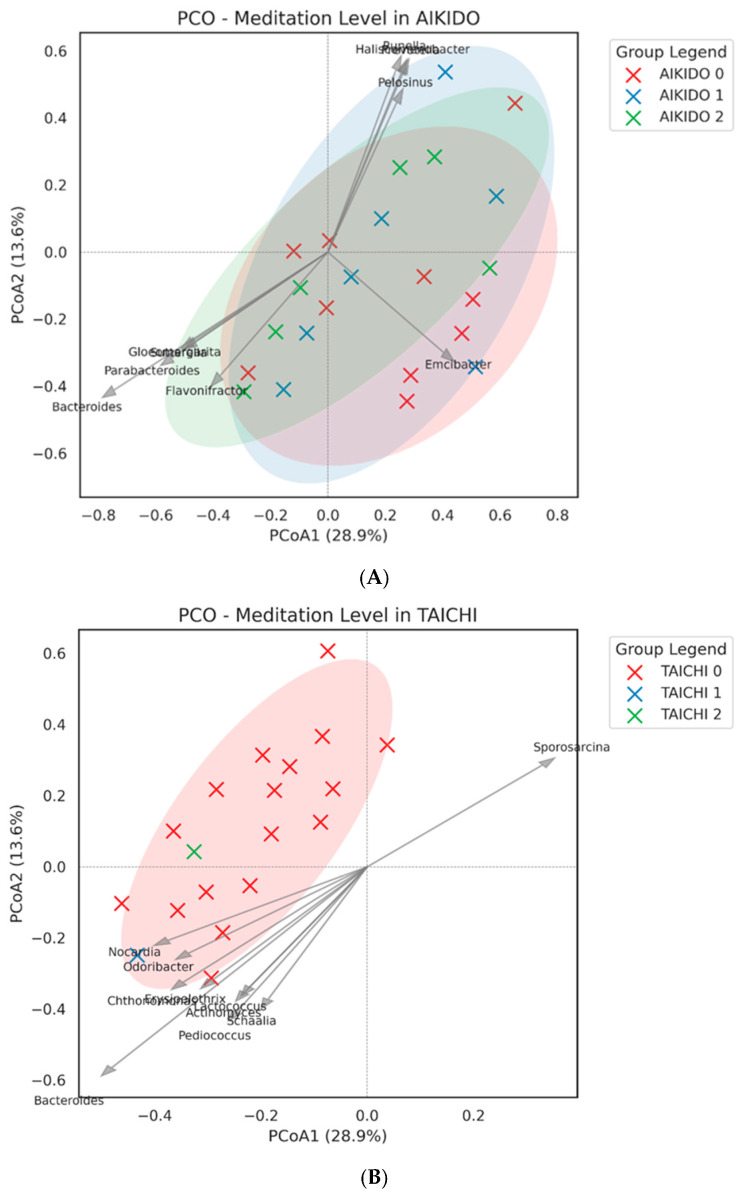
(**A**). Beta diversity by meditation level in Aikido. PCoA biplots of Bray–Curtis distances illustrating clustering of microbial communities across low, medium, and high meditation subgroups, with Bacteroides and Parabacteroides contributing to variation. (**B**). Beta diversity by meditation level in Tai Chi. PCoA biplots showing overlapping clusters with some differentiation between non-meditators and higher meditation groups, with taxa such as Sporosarcina and Odoribacter influencing ordination.

PCoA plots categorized by meditation levels (Low, Medium, High) within the TaiChi and Aikido groups (Figure 5A,B) exhibited observable patterns of variation: Participants with Low, Medium, and High meditation levels displayed distinct yet overlapping clusters in both groups. In the Aikido group (Figure 5A), a more gradual distinction in microbial communities across meditation levels 0, 1, and 2 was found, with some overlap between the groups. However, a slight trend toward differentiation in communities along the PCoA axes was noticeable. The first two axes similarly accounted for 28.9% and 13.6% of the variance. Important genera contributing to this distinction included *Bacteroides*, *Parabacteroides*, *Flavonifractor*, and *Pelosinus*, which could indicate functional microbial adaptations associated with meditation practice in Aikido. In the Taichi group (Figure 5B), the PCO indicated a partial separation of samples based on meditation level. Samples from meditation level 0 (those not engaging in meditation, marked in red) formed a distinct and tightly clustered group, while samples from higher meditation levels (levels 1 and 2) appeared more spread out among the first two principal coordinates, accounting for 28.9% and 13.6% of the total variance, respectively. In the Tai Chi group (Figure 5B), the PCoA indicated a partial separation of samples according to meditation level. Importantly, vectors for taxa such as *Sporosarcina*, *Odoribacter*, and *Bacteroides* significantly influenced the ordination space, underscoring specific microbial changes linked to meditation level. These findings were corroborated by ANOSIM analyses. Overall, meditation level significantly influenced gut microbial beta diversity across the entire cohort (R = 0.191, *p* = 0.035). Additionally, a strong interaction between sport and meditation was identified (R = 0.296, *p* = 0.001), implying that the effect of meditation level on microbiota composition may differ between Taichi and Aikido practices.

**Figure 6:**
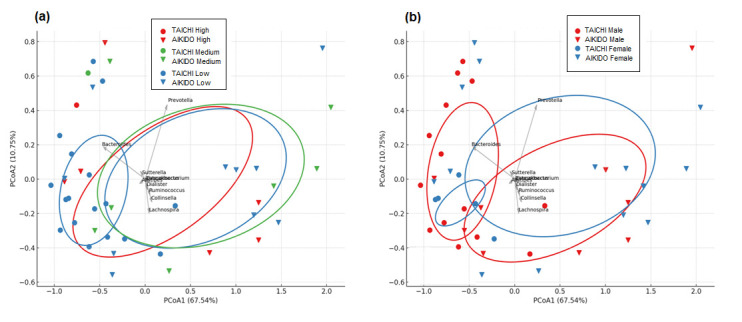
Beta diversity by meditation level and gender. (**a**). PCoA biplots combining meditation scores with sports category. (**b**). PCoA biplots combining meditation scores and gender, indicating partial clustering of samples with Prevotella and Bacteroides as key taxa.

The combined effect of meditation levels in taichi and aikido together shows the leading taxa, such as *Prevotella*, *Bacteroides*, *Ruminococcus*, and *Collinsella*, were responsible for this variation, with *Prevotella* typically linked to higher PCoA1 values and *Bacteroides* connected to lower PCoA1 values. At the genus level, taxa like *Prevotella* and *Bacteroides* continued to be major contributors to the variation observed along the PCoA axes. PCoA1 and PCoA2 accounted for 67.54% and 10.75% of the total variance (Figure 6a), respectively, consistent with genus-level patterns showing a good consistency. Moreover, a Spearman correlation analysis conducted between the MEDI score and the relative abundance of important bacterial genera did not show any significant relationships (*p* > 0.05), the spread of the ellipses implied that meditation levels might affect microbiota structure, although the separation was not absolute. In both Aikido and TaiChi participants (Figure 6b), gender-based PCoA biplots indicated a degree of separation between male and female participants: The first two PCoA axes accounted for 67.54% (PCoA1) and 10.75% (PCoA2) of the variance. PCoA biplots indicated some degree of gender-based separation, with partial clustering of male and female samples, particularly along PCoA1. In both groups, *Prevotella* and *Bacteroides* were the primary genera that contributed to the variation along PCoA1. Other genera, including *Ruminococcus*, *Collinsella*, and *Dialister*, also played a role in the distribution of samples. Ellipses illustrated some differences in clustering between male and female participants, though there were also overlaps, suggesting a minimal impact of gender on microbiota composition. While gender appeared to contribute modestly to microbiota composition, the observed differences were not absolute; confidence ellipses for male and female participants overlapped substantially. These findings suggest that gender might play a secondary role relative to the type of physical activity practice Aikido vs. Tai Chi in shaping gut microbiota in this cohort.

### 3.5. Genus & Phylum Level Microbiota Composition and Differences

**Figure 7:**
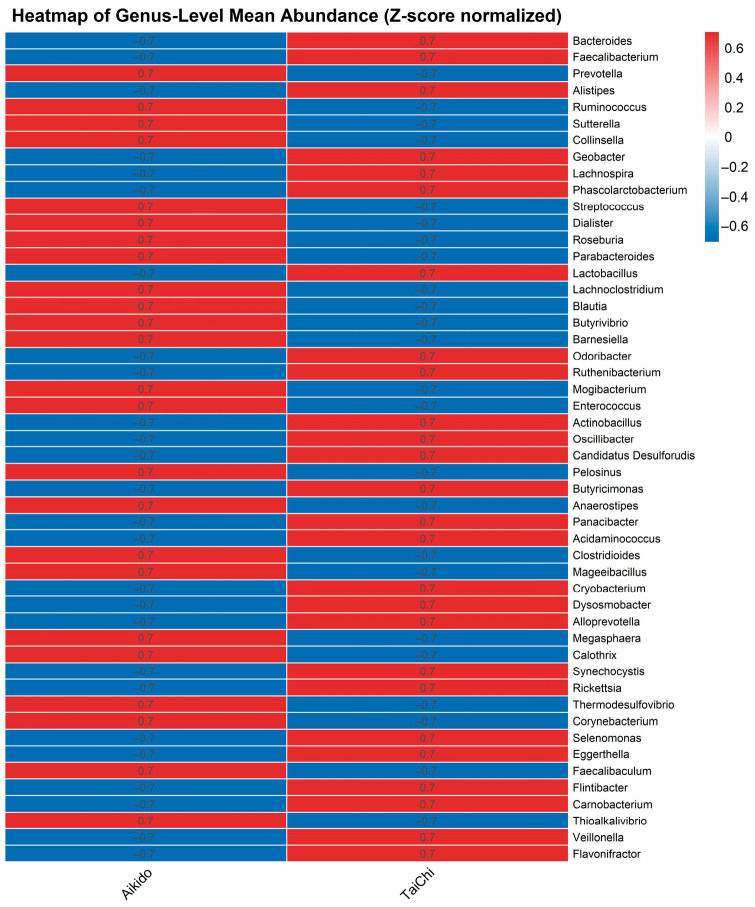
Genus-level abundance heatmap. Heatmap of normalized mean abundance (Z-scores) across 50 most abundant bacterial genera in Tai Chi and Aikido participants. Differences included *higher Faecalibacterium* and *Roseburia* in Aikido and greater *Collinsella* in Tai Chi.

A heatmap of genus-level mean abundance (normalized using Z-scores) was created to assess the differences in gut microbiota composition between the Aikido and Tai Chi groups, among the 503 genera detected and the top 50 most abundant genera were visualized in the heatmap (Figure 7). The heatmap featured a diverse array of bacterial genera, including significant taxa such as *Bacteroides*, *Faecalibacterium*, *Prevotella*, *Ruminococcus*, *Alistipes*, *Sutterella*, *Collinsella*, *Roseburia*, *Blautia*, and *Lactobacillus*, among others. Distinct patterns of genus abundance were identified between the groups: certain genera exhibited greater relative abundance in one group as opposed to the other (positive versus negative Z-scores). For instance, genera like *Bacteroides*, *Faecalibacterium*, *Prevotella*, *Ruminococcus*, and *Alistipes* generally demonstrated increased abundance in one group, whereas genera such as *Lactobacillus*, *Barnesiella*, and *Collinsella* were found to be more abundant in the opposing group. Z-score normalization facilitated the visualization of relative abundance differences regardless of the actual abundance levels. The vertical axis displays the genera, while the horizontal axis represents the Aikido and Tai Chi groups. The color intensity denotes the Z-score of the mean genus abundance. Shades of red (positive Z-scores) signify a higher-than-average abundance in the respective group, whereas shades of blue (negative Z-scores) indicate a lower-than-average abundance. A Z-score of 0 reflects the average abundance across both groups. To evaluate the differences in gut microbiota composition at the genus level between practitioners of Aikido and Tai Chi, a non-parametric Mann–Whitney U test was conducted on the normalized relative abundances of each identified genus. The resulting *p*-values were adjusted for multiple comparisons using the Benjamini–Hochberg false discovery rate (FDR) procedure. Among the 503 genera that were assessed, five displayed statistically significant differences between the two groups (FDR < 0.05): *Thioalkalivibrio*, *Pediococcus*, *Faecalibaculum*, *Butyrivibrio*, and *Rhodanobacter*. It is important that *Thioalkalivibrio*, *Pediococcus*, and *Faecalibaculum* showed highly significant disparities (FDR-adjusted *p* < 1.0 × 10^−6^).

**Figure 8:**
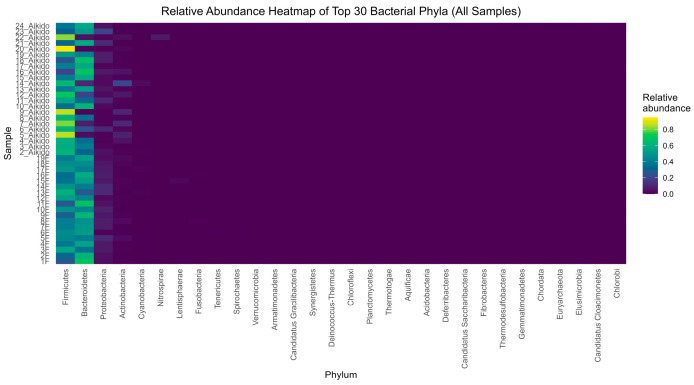
Phylum-level abundance heatmap and significant phyla. Heatmap of Z-score normalized phylum abundances, dominated by Firmicutes and Bacteroidetes.

Additionally, a heatmap of phylum-level mean abundance (normalized using Z-scores) was generated to examine the gut microbiota composition between the Aikido and Tai Chi groups (Figure 8). This normalization enables the comparison of patterns among genera with varying absolute abundances, and a total of 30 phyla detected. Overall, the heatmap underscores the compositional differences in gut microbiota profiles of the Aikido and Tai Chi participants. Overall *Firmicutes* and *Bacteroidetes* dominate both groups, but Aikido samples show a stronger relative abundance (brighter signal) of *Firmicutes*, *Bacteroidetes*, and *Proteobacteria* while Taichi samples have a generally lower and more uniform profile. Other phyla (many on the right of the heatmap) are essentially absent (dark purple) in both groups. At the phylum level, five phyla displayed statistically significant differences between the groups (FDR < 0.05): *Armatimonadetes*, *Candidatus Gracilibacteria*, *Planctomycetes*, *Nitrospirae*, and *Fusobacteria*. Notably, *Armatimonadetes* and *Candidatus Gracilibacteria* revealed highly significant differences (FDR-adjusted *p* < 1.0 × 10^−4^), indicating distinct ecological patterns associated with the two movement practices.

**Figure 9:**
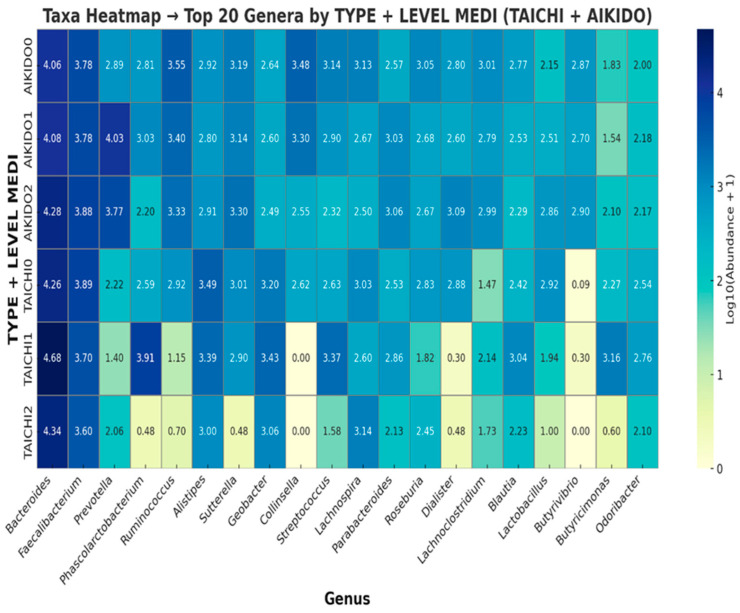
Heatmap of top 20 abundant genera by sport and meditation level. Visualization of community shifts with higher *Faecalibacterium*, *Roseburia*, and *Butyricimonas* in Aikido at higher meditation, and *Odoribacter* and Parabacteroides in Tai Chi at higher meditation.

These results are consistent with the visual patterns observed in the heatmaps at both genus and phylum levels, indicating that Aikido and Tai Chi practitioners exhibit distinct gut microbial compositions across taxonomic tiers. To further explore the taxonomic differences underlying the observed beta-diversity patterns, a heatmap was generated displaying the 20 most prevalent genera across combined sport type and meditation level groups (Figure 9). Overall, the microbial community composition showed variations associated with both the type of practice and meditation level. *Bacteroides* and *Faecalibacterium* were among the most abundant genera across all groups, in line with previous reports describing their widespread presence in the human gut microbiota. Within Aikido practitioners, differences in relative abundance of *Faecalibacterium*, *Roseburia*, *Ruminococcus*, and *Butyricimonas* were descriptively observed across meditation levels. As *Faecalibacterium* and *Roseburia* have been previously associated with short-chain fatty acid production, these taxonomic patterns may warrant further functional investigation; however, no functional or metabolomic measurements were performed in the present study. Conversely, *Alistipes* and *Sutterella* displayed lower relative abundances in higher meditation-level Aikido groups. In Tai Chi practitioners, similar trends were observed, with higher meditation levels showing increased relative abundances of *Faecalibacterium* and *Roseburia*, alongside differences in other taxa when compared with Aikido. For example, *Odoribacter* and *Parabacteroides* were more abundant in higher meditation-level Tai Chi groups than in the highest meditation-level Aikido group. Taken together, these findings suggest that both practice type and meditation level are associated with distinct taxonomic profiles of the gut microbiota. These observations are consistent with beta-diversity patterns observed in PCoA and ANOSIM analyses and should be interpreted as descriptive and hypothesis-generating rather than indicative of specific functional effects. Given the extreme imbalance of meditation-level strata within sport groups, including single-participant cells, interaction analyses should be interpreted exclusively as exploratory signals and not as evidence of differential effects.

### 3.6. Phylogenetic Cladogram of Differentially Abundant Taxa Across Aikido and Tai Chi

**Figure 10:**
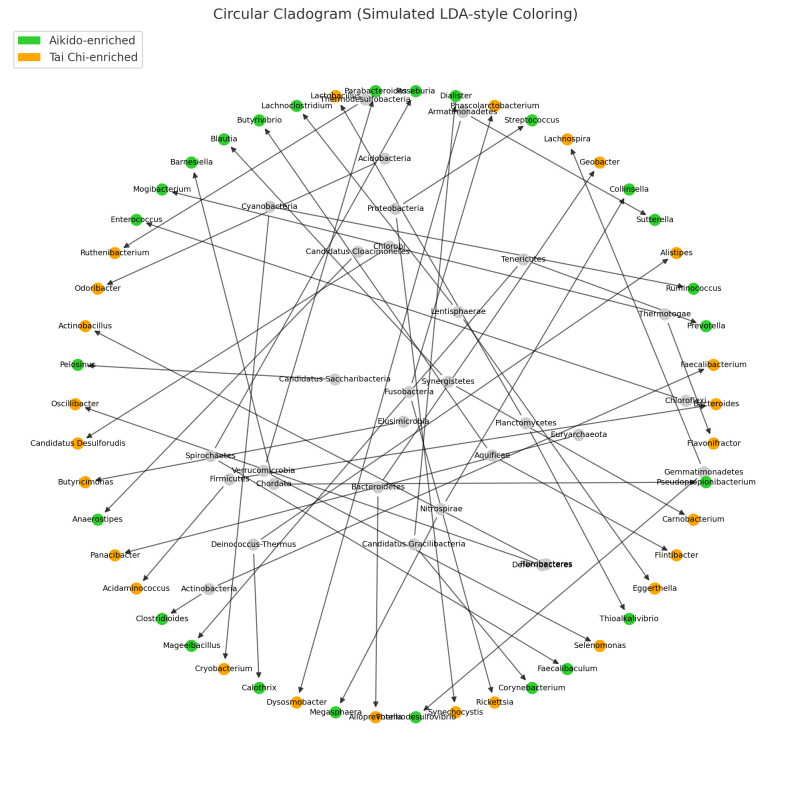
Phylogenetic cladogram of top 50 genera. Circular cladograms show phylogenetic relationships, with green nodes indicating genera enriched in Aikido (e.g., *Faecalibacterium, Roseburia*) and orange nodes indicating genera enriched in Tai Chi (e.g., *Prevotella*, *Collinsella*).

A differential abundance analysis was conducted to assess the gut microbiota composition between participants of Aikido and Tai Chi. The circular cladogram depicts the phylogenetic relationships of the fifty most abundant genera. The colors of the nodes indicate group-specific enrichment; green nodes signify genera that are enriched in the Aikido group, while orange nodes represent those enriched in the Tai Chi group (Figure 10). Genera found to be enriched in Aikido included members of the *Firmicutes* and *Bacteroidetes* phyla, such as *Faecalibacterium*, *Ruminococcus*, *Roseburia*, *Bacteroides*, and *Barnesiella*. In contrast, genera enriched in Tai Chi included *Prevotella*, *Collinsella*, *Lactobacillus*, *Dialister*, and *Phascolarctobacterium.*

**Figure 11:**
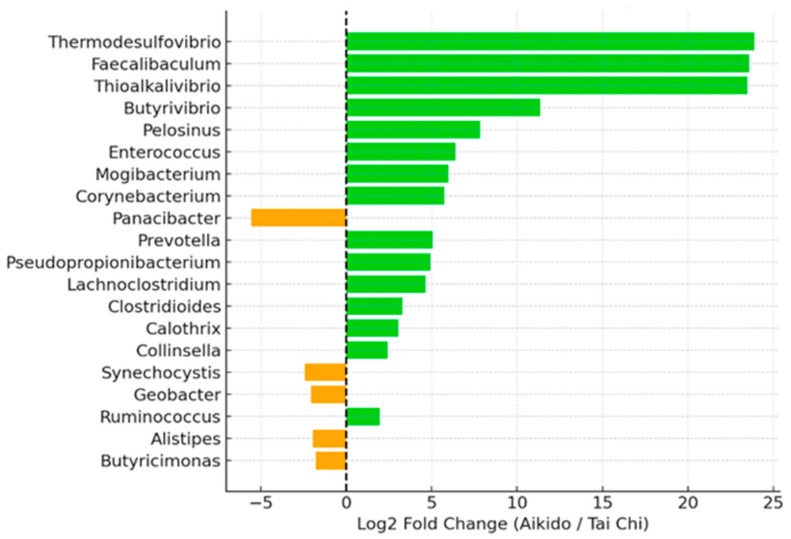
Differential abundance bar plot. Bar plot of the top 20 genera showing the largest log2 fold changes between groups, with *Faecalibacterium* and *Roseburia* enriched in Aikido and *Prevotella* and *Collinsella* enriched in Tai Chi.

The bar plot depicts the top twenty genera with the greatest log2 fold change between the two groups: genera such as *Faecalibacterium* and *Roseburia* showed higher abundance in Aikido, while *Prevotella* and *Collinsella* were more abundant in Tai Chi. These findings indicate that participants of Aikido and Tai Chi exhibit distinct profiles in gut microbiota, highlighting significant variations in the abundance of several key genera. Such differences may reflect variations in lifestyle, dietary patterns, or physiological impacts related to these two movement disciplines (Figure 11).

## 4. Discussion

This study was designed as an exploratory, cross-sectional investigation aimed at characterizing gut microbiota profiles within a specific population of non-competitive meditative martial arts practitioners. To our knowledge, this is among the first studies to systematically examine and compare gut microbiome characteristics in practitioners of different meditative movement styles, while also considering meditation depth and gender as relevant analytical dimensions. Accordingly, the findings should be interpreted as associative and hypothesis-generating rather than causal. Observed differences related to practice type and meditation depth reflect within-cohort variability and do not allow attribution of microbiota changes to Tai Chi, Aikido, or meditation per se. Our results indicate that while overall alpha diversity (Shannon and Simpson indices) was comparable across groups (Figure 2), beta-diversity analyses revealed distinct compositional patterns in gut microbiota between Aikido and Tai Chi practitioners (Figure 3 and Figure 4). These findings are consistent with previous evidence suggesting that the qualitative characteristics of physical activity—beyond intensity or duration—may influence gut microbial community structure [5,57,64,65,66,67]. Recent evidence supports the notion that differences in exercise modality and movement quality are associated with gut microbiota variation through pathways related to immune activation, gut permeability, and microbial metabolism [5,67,68].

Surprisingly, although Tai Chi is typically seen as a gentler, lower-intensity practice compared to Aikido [30,31,32,33] our findings reveal that both disciplines cultivate unique gut microbiota profiles. This is in line with recent research suggesting that elements such as movement style, breath control, and mind–body synchronization have distinct effects on the gut microbiome, irrespective of basic exercise volume [69,70]. The impact of meditation depth on gut microbiota composition was examined through PCoA clustering and Spearman correlation analysis (Figure 5). Although minor clustering by meditation level was observed, no significant correlation was identified between MEDI score and key bacterial genera. This is consistent with growing evidence that, while mindfulness and meditation can affect gut–brain axis function and stress-related physiology [6,13], their direct influence on gut microbiota composition may necessitate longer or more intensive interventions to become evident [66,67,68]. It is conceivable that meditation has a more substantial impact on microbial function (e.g., metabolite synthesis) rather than just on taxonomic composition.

Our investigation suggests that both meditation depth and the specific type of meditative movement practice (Tai Chi or Aikido) were associated with differences in gut microbiota composition within this group of practitioners. Beta-diversity patterns, illustrated through Principal Coordinates Analysis (PCoA) based on Bray–Curtis distances, revealed compositional variation of gut microbial communities across meditation levels and between the two practices. The first two PCoA axes accounted for 56.85% and 15.78% of the overall variance, respectively. While the type of sport alone did not show a significant effect on beta diversity, the level of meditation was significantly influential (ANOSIM R = 0.191, *p* = 0.035). An exploratory interaction signal between sport type and meditation level was also observed (ANOSIM R = 0.296, *p* = 0.001). However, given the small and imbalanced size of certain meditation-level subgroups, this finding should be interpreted with caution and considered hypothesis-generating rather than confirmatory. In analyzing PCoA by meditation level within each sport, a clear differentiation of communities was observed, particularly within the Tai Chi group (Figure 5B). Here, individuals who did not meditate (level 0) formed a distinct cluster, whereas those with higher levels of meditation (levels 1 and 2) were more scattered across the first two principal coordinates. Significant taxa contributing to this differentiation included *Sporosarcina, Odoribacter*, and *Bacteroides* (Figure 6). In contrast, Aikido practitioners exhibited a more gradual transition in community structure as meditation levels increased (Figure 5A), with genera such as *Bacteroides*, *Parabacteroides*, *Flavonifractor,* and *Pelosinus* influencing the observed variation. At the genus level, a heatmap of mean relative abundance revealed distinct microbial signatures between Aikido and Tai Chi practitioners (Figure 7). Among the 503 genera identified, five showed statistically significant differences between groups after FDR correction (FDR < 0.05): *Thioalkalivibrio*, *Pediococcus*, *Faecalibaculum*, *Butyrivibrio*, and *Rhodanobacter*. Additional genera, including *Bacteroides*, *Faecalibacterium*, *Prevotella*, *Ruminococcus*, and *Alistipes*, displayed group-specific distribution patterns [67,68]. SCFA-associated taxa such as *Faecalibacterium* and *Roseburia* were relatively more abundant in Aikido practitioners with higher meditation levels. Although these taxa have been linked in previous studies to gut barrier integrity and immune modulation, no functional, metagenomic, or metabolomic assessments were performed in the present study. Consequently, any inferred links to SCFA production or gut–brain axis pathways remain speculative and are proposed solely as hypotheses for future investigation [71,72,73].

In contrast, Tai Chi practitioners exhibited relatively higher abundances of *Prevotella* and *Collinsella*. At the phylum level, five phyla differed between groups after false discovery rate correction (FDR < 0.05): Armatimonadetes, Candidatus Gracilibacteria, Planctomycetes, Nitrospirae, and Fusobacteria (Figure 8). Overall, Firmicutes and Bacteroidetes predominated in both groups, although their relative abundances varied according to practice type and meditation level. These less-characterized phyla have been previously linked to host–microbe interactions, redox metabolism, and immune-related processes [73,74,75].

Phylogenetic representation of the top 50 genera further illustrated compositional differences between practices (Figure 10). Genera relatively enriched in Aikido included *Faecalibacterium*, *Ruminococcus*, *Roseburia*, *Bacteroides*, and *Barnesiella*, whereas Tai Chi practitioners showed enrichment of *Prevotella*, *Collinsella*, *Lactobacillus*, and *Dialister*. Consistently, bar plots of the top 20 differentially abundant genera highlighted practice-specific distribution patterns (Figure 11).

SCFA-associated genera such as *Faecalibacterium* and *Roseburia* were relatively more abundant in Aikido practitioners with higher meditation levels (Figure 9). Similarly, Tai Chi practitioners at higher meditation levels displayed enrichment of *Odoribacter* and *Parabacteroides*, taxa previously associated with metabolic and immune-related pathways [57,76,77,78]. However, no functional, metagenomic, or metabolomic measurements were conducted in the present study, and therefore these taxonomic patterns cannot be directly linked to SCFA production or gut–brain axis signaling.

Taken together, these findings indicate that gut microbiota composition differed according to meditative movement practice and meditation depth within this cohort. These differences were primarily reflected in beta-diversity patterns and taxonomic distributions, while alpha diversity remained largely comparable across groups. Given the observational design, single time-point sampling, and small and imbalanced subgroups, the present results should be interpreted as descriptive and hypothesis-generating.

Several limitations of the present study should be acknowledged. First, the cross-sectional and observational design precludes any inference of causality between meditative movement practices, meditation depth, and gut microbiota composition. Second, the absence of a sedentary control group or practitioners of non-mind–body physical activities limits the ability to attribute observed microbiota differences specifically to Tai Chi, Aikido, or meditation-related components. The overall sample size was modest, and certain subgroup analyses—particularly those stratified by meditation level within each practice—were characterized by small and imbalanced group sizes. As a result, interaction effects should be interpreted with caution and considered exploratory and hypothesis-generating rather than confirmatory. In addition, gut microbiota profiling was based on a single fecal sample per participant, which does not account for intra-individual temporal variability. The use of a 16S rRNA OTU-based analytical pipeline and the lack of functional, metagenomic, metabolomic, or clinical measurements further limit mechanistic interpretation of the observed taxonomic patterns. Consequently, potential links to short-chain fatty acid production, immune modulation, or gut–brain axis signaling cannot be directly assessed. Finally, although several lifestyle and demographic variables were collected, residual confounding cannot be excluded. Taken together, these limitations highlight the exploratory nature of the present work and underscore the need for future longitudinal, adequately powered studies incorporating functional microbiome analyses and clinical or psychological outcomes.

Future longitudinal studies incorporating functional microbiome profiling, metabolomics, and clinical or psychological outcomes will be necessary to clarify the biological relevance and potential mechanisms underlying the observed associations.

## Figures and Tables

**Table 1:** Socio-demographic characteristics of Tai Chi and Aikido participants.

Variable	Value Aikido	Value Taichi	*p*-Value
Gender			0.03
female	9 (39.13%)	14 (73.68%)	
male	14 (60.87%)	5 (26.31%)	
Age			0.29
18–30	1 (4.17%)	1 (5.26%)	
31–65	20 (83.33%)	12 (63.16%)	
>65	3 (12.5%)	6 (31.58%)	
Educational level			0.36
Middle school graduation	1 (4.17%)	0 (0.00%)	
High school degree	9 (37.50%)	6 (40.00%)	
Bachelor’s degree	1 (4.17%)	2 (13.33%)	
Master’s degree	9 (37.50%)	7 (46.67%)	
PhD	4 (16.67%)	0 (0.00%)	
Smoking			0.74
No	16 (66.67%)	14 (73.68%)	
Yes	8 (33.33%)	5 (26.32%)	
Smoking frequency			0.42
At least once/day	6 (75.00%)	2 (40.00%)	
4–6 times/week	1 (12.50%)	2 (40.00%)	
Maximum 3 times/week	1 (12.50%)	1 (20.00%)	
BMI			0.50
Normal weight	17 (70.83%)	12 (63.16%)	
Underweight	0 (0.00%)	1 (5.26%)	
Overweight	6 (25.00%)	6 (31.58%)	
Obese	1 (4.17%)	0 (0.00%)	
Diet			0.3
No specific diet	10 (41.67%)	5 (26.32%)	
Mediterranean	8 (33.33%)	12 (63.16%)	
Vegetarian	3 (12.50%)	1 (5.26%)	
Requested by physician	1 (4.17%)	0 (0.00%)	
Caloric restriction	1 (4.17%)	0 (0.00%)	
Gluten free	0 (0.00%)	1 (5.26%)	
Other	1 (4.17%)	0 (0.00%)	
Alcohol consumption			0.20
No alcohol	0 (0.00%)	3 (15.79%)	
Often (>5 times/month)	11 (45.83%)	6 (31.58%)	
Sometimes (3–4 times/month)	10 (41.67%)	7 (36.84%)	
Occasionally (1–2 times/month)	3 (12.50%)	3 (15.79%)	
Aikido practice			0.50
<300 min/week	14 (58.33%)	9 (47.37%)	
>300 min/week	10 (41.67%)	10 (52.63%)	
General physical activity			0.29
Partially active	4 (16.67%)	6 (31.58%)	
Physically active	20 (83.33%)	13 (68.42%)	
MEDI score			0.005
Low (0–60)	10 (41.67%)	17 (89.47%)	
Medium (60–80)	7 (29.17%)	1 (5.26%)	
High (80–120)	7 (29.17%)	1 (5.26%)	
Meditation time			0.13
<60 min/week	13 (54.17%)	6 (31.58%)	
>60 min/week	11 (45.83%)	11 (57.89%)	
Not available	0 (0.00%)	2 (10.53%)	

## Data Availability

Data can be obtained from the corresponding author upon reasonable request. All sequencing data generated within the project have been deposited in the Sequence Read Archive (SRA) under BioProject accession number PRJNA1311479.

## Supplementary Materials

The following supporting information can be downloaded at: https://www.mdpi.com/article/10.3390/microorganisms14020275/s1; Table S1: ANOSIM results for gut microbiota composition.

## Abbreviations

WHO	World Health Organization
SCFA	Short-Chain Fatty Acid
BMI	Body Mass Index
MET	Metabolic Equivalent of Task
MEDEQ	Meditation Depth Questionnaire
MEDI	Meditation Depth Index
OTU	Operational Taxonomic Unit
PCR	Polymerase Chain Reaction
PCoA	Principal Coordinates Analysis
ANOSIM	Analysis of Similarities
PA	Physical Activity
FDR	False Discovery Rate
SPSS	Statistical Package for the Social Sciences
IPAQ	International Physical Activity Questionnaire
DNA	Deoxyribonucleic Acid
RDP	Ribosomal Database Project
SD	Standard Deviation
