# Supplementary material for: Meditation in Motion: Sport Type and Meditation Level Shape Gut Microbiota Profiles in Aikido and Tai Chi Practitioners
Source: Microorganisms. 2026 Jan 24;14(2):275. doi: 10.3390/microorganisms14020275 (PMC12943227; doi:10.3390/microorganisms14020275)
Supplement: Supplementary file 1 [file microorganisms-14-00275-s001.zip › microorganisms-4018457-supplementary.pdf]

Table S1: ANOSIM results for gut microbiota composition. R statistics and p-values for comparisons across BMI, gender, physical activity, meditation level, and their interactions with sport type. Significant effects were observed for meditation level and for the combined sport + meditation interaction, while BMI and most other factors showed no differences

Factor	ANOSIM R	p-value (%)	Interpretation
BMI	-0.008	49.40%	No difference
Gender	0.015	22.80%	No difference
PA level	-0.019	70.20%	No difference
MEDI level	0.191	3.50%	Significant
BMI (Men)	-0.125	94.80%	No difference
MEDI (Men)	0.211	5.70%	Significant
Sport type (Men)	-0.127	83.30%	No difference
BMI (Women)	0.068	20%	No difference
MEDI (Women)	0.24	7.80%	Borderline significant
PA (Women)	-0.078	86.10%	No difference
Sport + BMI interaction	0.064	17.40%	No difference
Sport + Gender interaction	0.091	4.90%	Significant
Sport + MEDI interaction	0.296	0.10%	Highly significant
Sport + PA interaction	0.049	11.60%	No difference
